# Bone marrow mesenchymal stem cells suppress metastatic tumor development in mouse by modulating immune system

**DOI:** 10.1186/s13287-015-0039-8

**Published:** 2015-03-24

**Authors:** Lei Zhang, Xiao San Su, Jun Song Ye, Yi Yin Wang, Zheng Guan, Yan Feng Yin

**Affiliations:** Biomedical Research Center, Affiliated Calmette Hospital of Kunming Medical University, 504 Qing Nian Road, Kunming, Yunnan 650011 PR China

## Abstract

**Introduction:**

Bone marrow mesenchymal stem cells (BMSCs) have been studied extensively because of their potential use in clinical therapy, regenerative medicine, and tissue engineering. However, their application in tumor therapy remains yet in preclinical stage because of the distinct results from different researches and vagueness of their functional mechanism. In this study, the influence of BMSCs on tumor growth was observed and the potential mechanism was investigated.

**Method:**

Two animal models, H22 ascitogenous hepatoma in BALb/c mouse and B16-F10 pulmonary metastatic melanoma in C57 mouse, were adopted in experience *in vivo* and treated with BMSCs by intravenous injection. The percentage of Gr-1^+^CD11b^+^ myeloid-derived suppressor cells (MDSCs) and IFN-γ^+^ T cells were observed in peripheral blood (PB) and bone marrow (BM) by Flow Cytometry. BMSCs were co-cultured *in vitro* with tumor cells and MDSCs in a tumor conditioned medium separately in order to illustrate the mechanism.

**Results:**

Our results demonstrated that BMSCs treatment caused a delayed tumor growth and a prolonged survival in both tumor models, the homing fraction of BMSCs in BM was 2% - 5% in 24–72 hours after transfusion and the percentage of Gr-1^+^CD11b^+^ MDSCs was downregulated in peripheral blood and BM. Meanwhile, IFN-γ^+^ T lymphocytes in PB increased. *In vitro* co-culture showed that BMSCs inhibited the induction and proliferation of MDSCs in tumor conditioned medium, whereas they didn’t affect the proliferation of B16-F10 and H22 cells by *in vitro* co-culture. Both *in vivo* and *in vitro* results showed that BMSCs have a systemic suppressive effect on MDSCs.

**Conclusion:**

Our data suggest that BMSCs has suppressive effect on tumor and is feasible to be applied in cancer treatment. BMSCs inhibiting MDSCs induction and proliferation is likely one of the mechanism.

## Introduction

Owing to their multiple differentiation capacities and their immune modulation effect, bone marrow mesenchymal stem cells (BMSCs) have been widely used in regeneration of tissue such as bone [1], cartilage [2], liver [3], cardiovascular repair [4], and cell therapy in autoimmune disease [5] since they were discovered in 1999 [6]. In recent years, mesenchymal stem cells (MSCs) have received intensive attention in the field of tumors owing to their tumor tropism [7], angiogenesis [8], and immune modulation [9]. Research on application of MSCs mainly focuses on two fields. Some investigators take BMSCs as attractive vehicles for delivering therapeutic agents such as the therapeutic gene P53 [10], oncolytic virus [11,12], anti-tumor chemotherapeutic drug [13], and special cell factors such as pigment epithelium-derived factor [14], interleukin-12 and interferon beta [15]. Other investigators established a variety of tumor models in which MSCs are introduced without modification and their impact on tumor development is evaluated. Studies have reported contradicting results, with some investigators finding that MSCs promote tumor growth and others reporting that MSCs inhibit tumor growth. Samaniegeo and colleagues identified three subsets of MSCs that contribute to regulate different steps of leukocyte tumor infiltration: CD90^+^ cells surrounding peritumoral vessels secrete C-C motif chemokine ligand CCL2 to recruit leukocytes at the tumor periphery, which inhibit development of malignant melanoma; intratumoral fibroblast activation protein FAP^+^ cells organize a stromal scaffold that contact guides further invasion among densely packed tumor cells; and CD90^+^FAP^+^ MSCs have no effects on tumor [16]. Bruno and colleagues found that microvesicles derived from human BMSCs inhibited cell cycle progression in several tumor cell lines. The microvesicles induced apoptosis in HepG2 and Kaposi's cells. They caused also necrosis in Skov-3 both *in vivo* and *in vitro* [17]. Gong and colleagues, however, found that BMSCs could promote the growth of hepatoma by improving microvascular formation [8].

The reason for these discrepancies is unknown, but they may be attributable to differences in tumor models, animal hosts, heterogeneity of MSCs, dose or timing of the MSCs injected, or other factors that are not yet appreciated. Despite all these extensive investigations over the past 10 years, the impact of MSCs on tumor progression remains unclear. The effects of BMSCs on tumor growth are mainly due to either MSC-producing factors within the tumor microenvironment or MSC-modulating immune cells, which have intrigued intensive studies intensively in recent years.

MSCs have been shown to directly suppress the function of a variety of immune cells, including T and B lymphocytes, dendritic cells and nature killer cells [18,19]. They can also recruit macrophages and granulocytes infiltrating into tumors, which may contribute to anti-tumor effects in the rat colon carcinoma model [20]. Myeloid-derived suppressor cells (MDSCs) are a heterogeneous cell population of myeloid origin and can be activated and expanded in response to growth factors and cytokines released by tumors. Once MDSCs are activated, they accumulate in lymphoid organs and tumors where they exert T-cell immunosuppression [21]. Whether MDSCs take part in the MSC suppression events and what role they may play have not been studied.

In this study, we would like to explore: firstly, the effects of BMSCs on H22 ascitogenous hepatoma in the BALb/c mouse and B16-F10 pulmonary metastatic melanoma in the C57 mouse; and, secondly, the potential mechanisms of MSC immune modulation action, particularly the interaction of BMSCs and MDSCs through the above two models.

## Materials and methods

### Tumor cell lines and mice

The following standard experimental cell lines were used *in vitro* and *in vivo*: B16-F10 (H-2^b^) melanoma, H22 hepatoma (H-2^d^), and NIH-3T3 murine fibroblasts. All cells were cultured in RPMI 1640 medium (Gibco-BRL, Carlsbad, CA, USA) supplemented with 10% fetal bovine serum (heat inactivated), penicillin (100 U/ml), and streptomycin (100 μg/ml). Inbred C57BL/6 (B6, H-2^b^) and BALb/c (H-2^d^) female mice (8 weeks) used in animal models were purchased from Beijing HFK Bioscience Co., Ltd (Beijing, China).

### Isolation and culture of bone marrow mesenchymal stem cells

BMSCs were isolated from male mouse, cultured, and characterized as described previously [22]. Mice were sacrificed by heart injection of potassium chloride, and then the femora and tibiae were isolated. After dissection of attached muscle and connective tissue from the bones, the marrow was flashed with Dulbecco’s modified Eagle’s medium (Gibco-BRL) through a needle stabbed into the epiphysial ends of the bones. Cells were then separated by Percoll (ρ = 1.077; Gibco-BRL) density gradient centrifugation, rinsed with Dulbecco’s modified Eagle’s medium twice, and cultured in Dulbecco’s modified Eagle’s medium with 10% heat-inactivated fetal bovine serum, penicillin (100 U/ml), and streptomycin (100 μg/ml) (BMSC medium). Afterwards, BMSCs were maintained in a humidified incubator (37°C, 5% carbon dioxide and 20% oxygen) and their medium was changed on the seventh day and renewed every 4 days. The 90% confluent BMSCs were harvested and passed at low density (200 cells/cm^2^) under subconfluent conditions to prevent cell differentiation. BMSCs at passage 3 were applied for in *in vitro* and *in vivo* assays.

### Cell proliferation assay

For quantification of tumor cells in the co-culture experiments, carboxy-fluorescein diacetate succinimidyl ester-labeled (Invitrogen, Carlsbad, CA, USA) B16-F10 and H22 cells were cultured either alone (2 × 10^5^ cells/well of six-well culture plates) or in the presence of syngeneic BMSCs or NIH-3T3 cells (ratio 1:1) for 48 hours. Labeling of the cells was performed according to the supplier’s recommendations. Cells were detached with 0.25% trypsin/ethylenediamine tetraacetic acid disodium salt and washed twice with phosphate-buffered saline (PBS). The cell pellet was then resuspended in 1 ml PBS and the labeled tumor cells were visualized using flow cytometry (Cytomics FC500™; Beckman Coulter, Fullerton, CA, USA).

### Tumor models and treatment protocol

For the B16-F10 experimental pulmonary metastatic model, 2 × 10^5^ B16-F10 tumor cells were injected via the tail vein of C57 BL/6 mice on day 0. On days 0, 7, and 14, the mice received syngeneic 5 × 10^5^ cells/mouse BMSC treatment. C57 mice injected with B16-F10 without BMSC treatment were served as the control. The mice were numbered and peripheral blood from five of them in each group was acquired every 4 days from the tail vein. Then, 35 days after B16-F10 injection, these five animals from each group were sacrificed and their bone marrows were analyzed by flow cytometry. Meanwhile, their lungs were resected and weighed. The survival time of the remaining mice was continuously monitored for at least 100 days. To establish the ascitogenous hepatoma tumor model for monitoring the effect of BMSCs on hepatoma growth, BALb/c mice were inoculated intraperitoneally with 5 × 10^5^ H22 cells. On days 0 and 7, the mice received syngeneic 5 × 10^5^ cells/mouse BMSC treatment. BALb/c mice injected with H22 cells but without BMSC treatment served as the control. Mouse body weight was calculated and the survival time of mice was continuously monitored. MDSCs and interferon gamma (IFNγ)-positive T cells in their peripheral blood and bone marrow were analyzed using the same method as for C57 mice. The surviving analysis was also applied in BALb/c mice. All animal experiments were performed according to the guidelines and protocols approved by the Institutional Animal Care and Use Committee at Kunming Medical University (Kunming, China).

### Generation of MDSCs from bone marrow progenitors *in vitro*

To generate tumor cell conditioned medium, subconfluent B16-F10 or H22 cells were kept in RPMI 1640 medium with a reduced (3%) serum concentration for 48 hours. After that time, supernatants were collected, aliquoted, and stored at −80°C until further use. Bone marrow cells (5 × 10^5^ cells) obtained from the femurs and tibias of C57 and BALb/c mice were cultured in RPMI 1640 medium, alone or in the presence of 10%, 30%, or 60% v/v tumor cell conditioned medium, or co-cultured with 1 × 10^5^ NIH-3T3 (used as a control to eliminate the influence of cell concentration) or syngeneic 1 × 10^5^ BMSCs. Twenty-four, 48, and 72 hours after culture, floating cells were gently collected and numerated using a TC10 automated cell counter (Bio-rad, Hercules, CA, USA). The percentage of Gr-1^+^CD11b^+^ MDSCs was analyzed by flow cytometry and the absolute number of Gr-1^+^CD11b^+^ MDSCs was calculated according to the following formula:

$$ \mathrm{Absolute}\ \mathrm{number}\ \mathrm{of}\ \mathrm{MDSCs}=\mathrm{total}\ \mathrm{number}\ \mathrm{of}\ \mathrm{cells}\ \mathrm{harvested}\ \mathrm{from}\ \mathrm{each}\ \mathrm{well}\times \mathrm{percent}\ \mathrm{of}\ \mathrm{G}\mathrm{r}\hbox{-} {1}^{+}\mathrm{C}\mathrm{D}11{\mathrm{b}}^{+}\mathrm{MDSCs}\ \left(\%\right) $$

### Effects of BMSCs and MDSCs on peripheral blood CD3^+^IFNγ^+^ T cells *in vitro*

Peripheral blood white cells (5 × 10^5^ cells) obtained from C57 and BALb/c mice were co-cultured with 1 × 10^5^ NIH-3T3 or syngeneic BMSCs in 60% v/v tumor supernatant culture medium with or without the presence of 1 × 10^5^ bone marrow suspension cells obtained from femurs and tibias of C57 and BALb/c mice. After 24, 48, and 72 hours, floating cells were gently collected and numerated. The percentage of CD3^+^IFNγ^+^ T cells in the suspension cells was analyzed by flow cytometry.

### Flow cytometric analysis

Antibodies employed in flow cytometry were fluorescein isothiocyanate-conjugated anti-mouse Ly-6G/Ly-6C (Gr-1; clone RB6-8C5), CD25 (clone 3C7), phycoerythrin-conjugated anti-mouse CD11b (clone M1/70), IFNγ (clone XMG1.2) and phycoerythrin-Cy7-conjugated anti-mouse CD3 (clone 17A2) purchased from Biolegend (San Diego, CA, USA). Peripheral blood mononuclear cells and bone marrow cells were collected from C57 or BALb/c mice through the tail vein and bone marrow, respectively. The cell suspensions were stained with fluorescein-conjugated anti-mouse CD3, CD11b, Gr-1, and IFNγ monoclonal antibody at room temperature for 30 minutes. After washing with PBS, the samples were fixed with PBS containing 1% paraformaldehyde and analyzed by flow cytometry.

### Statistical analyses

For comparison of individual time points, Student’s *t* test was used to compare means between the two groups. Analysis of variance was used for the comparisons among three or more groups. Kaplan–Meier survival analysis was introduced in animal survival studies. Differences were considered significant when *P* <0.05. Statistical analysis was performed using commercially available software (SPSS 17.0, SPSS-China, Beijing, P.R. China).

## Results

### Bone marrow mesenchymal stem cells inhibit the growth of tumor in metastatic models

**Figure 1:**
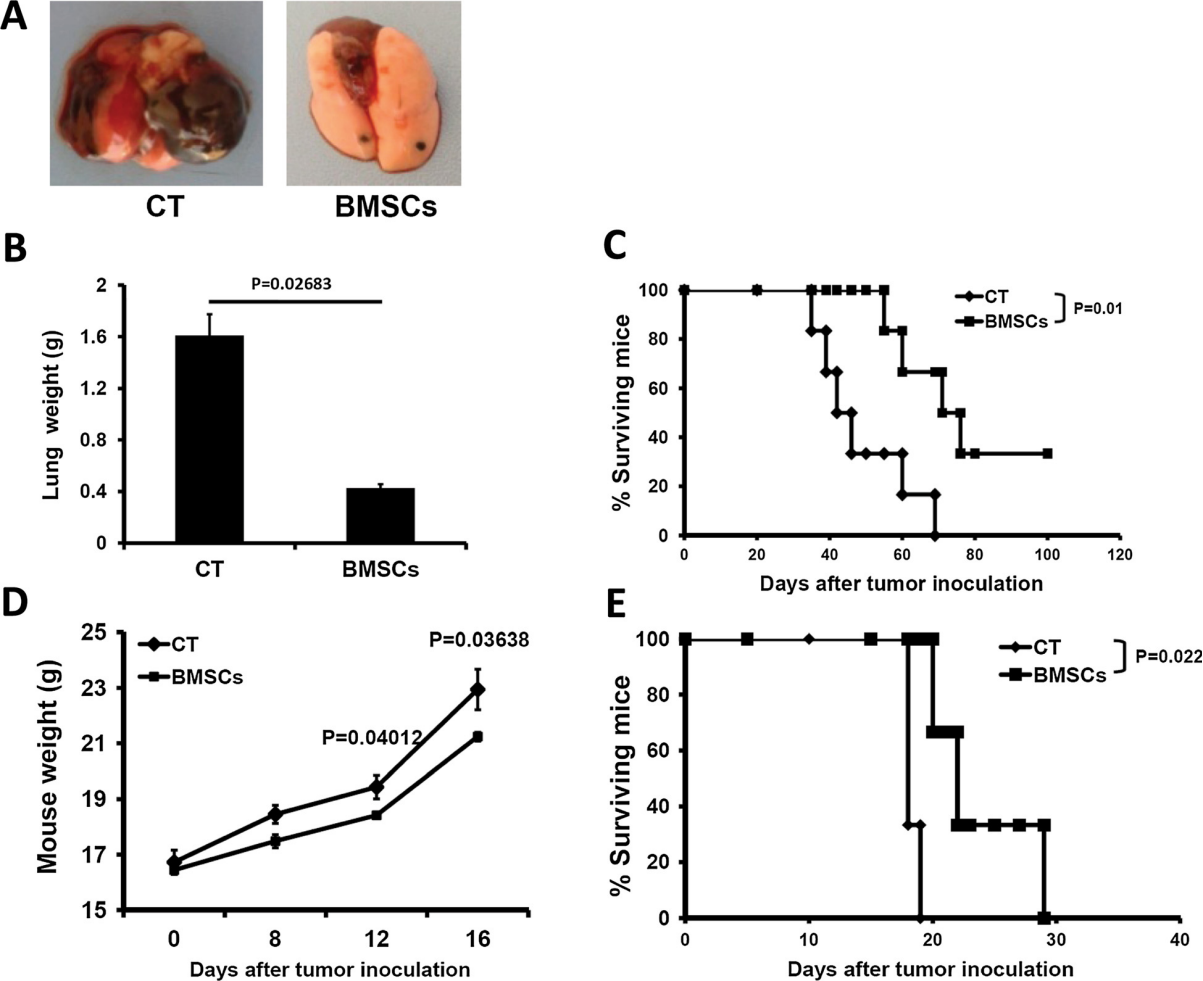
**Bone marrow mesenchymal stem cells inhibit the growth of metastatic tumor (** ***n*** **= 14).** Day 0, 2 × 10^5^ B16-F10 tumor cells were injected via the tail vein of C57 mice. On days 0, 7, and 14, the mice received syngeneic 5 × 10^5^ cells/mouse bone marrow mesenchymal stem cell (BMSC) treatment. C57 mice injected with B16-F10 without BMSC treatment served as the control. Thirty-five days after B16-F10 injection, five animals from each group were sacrificed and resected lungs were weighed. The survival time of the remaining mice was monitored. BALb/c mice were inoculated intraperitoneally with 5× 10^5^ H22 tumor cells. On days 0 and 7, the mice received syngeneic 5 × 10^5^ cells/mouse BMSC treatment. BALb/c mice injected with B16-F10 cells without BMSC treatment served as the control. Mouse body weight was calculated and the survival time of the mice was monitored continuously. **(A)** Example of tumor formation in a lung of the C57 mouse tumor model and BMSC-treated mouse. Less tumor was formed in the BMSC-treated mouse compared with the control group. **(B)** Lung weight of C57 mice. **(C)** Survival time of C57 mice. **(D)** Body weight of BALb/c mice. **(E)** Survival time of BALb/c mice. BMSCs, bone marrow mesenchymal stem cell-treated group; CT, control (tumor model) group.

The effects of intravenously injected mouse BMSCs on an artificial pulmonary metastatic mouse model are demonstrated in Figure 1A. The intravenous BMSC administration systemically inhibited the growth of metastatic lung tumors, as quantified by scoring the total lung weight in the mice inoculated with the B16-F10 tumor cells intravenously. The average lung weight of mice treated with BMSCs was 425 ± 30 mg, which was significantly lower than the lung weights for control mice (1,609 ± 166 mg), and the difference was statistically significant. The anti-tumor effect of BMSCs was further evaluated through mouse survival after intravenous injection of B16-F10 cells with or without BMSCs. Eventually, 33.3% of mice in the BMSC therapy group became long-term survivors (>100 days), as compared with none in the control animals (*P* = 0.01) (Figure 1B). In the ascitogenous hepatoma model, BALb/c mice received syngeneic BMSC treatment intravenously after H22 tumors were injected intraperitoneally. As shown in Figure 1C, the body weight of the animals inoculated with H22 cells and treated with BMSCs was significantly lower than those inoculated with H22 only on days 12 and 16, which indicates that BMSC treatment inhibited ascite formation. BMSC treatment also prolonged the survival time of H22 inoculated mice (*P* = 0.022) (Figure 1D).

### No effect of BMSCs on the proliferation of tumor cells cultured *in vitro*

**Figure 2:**
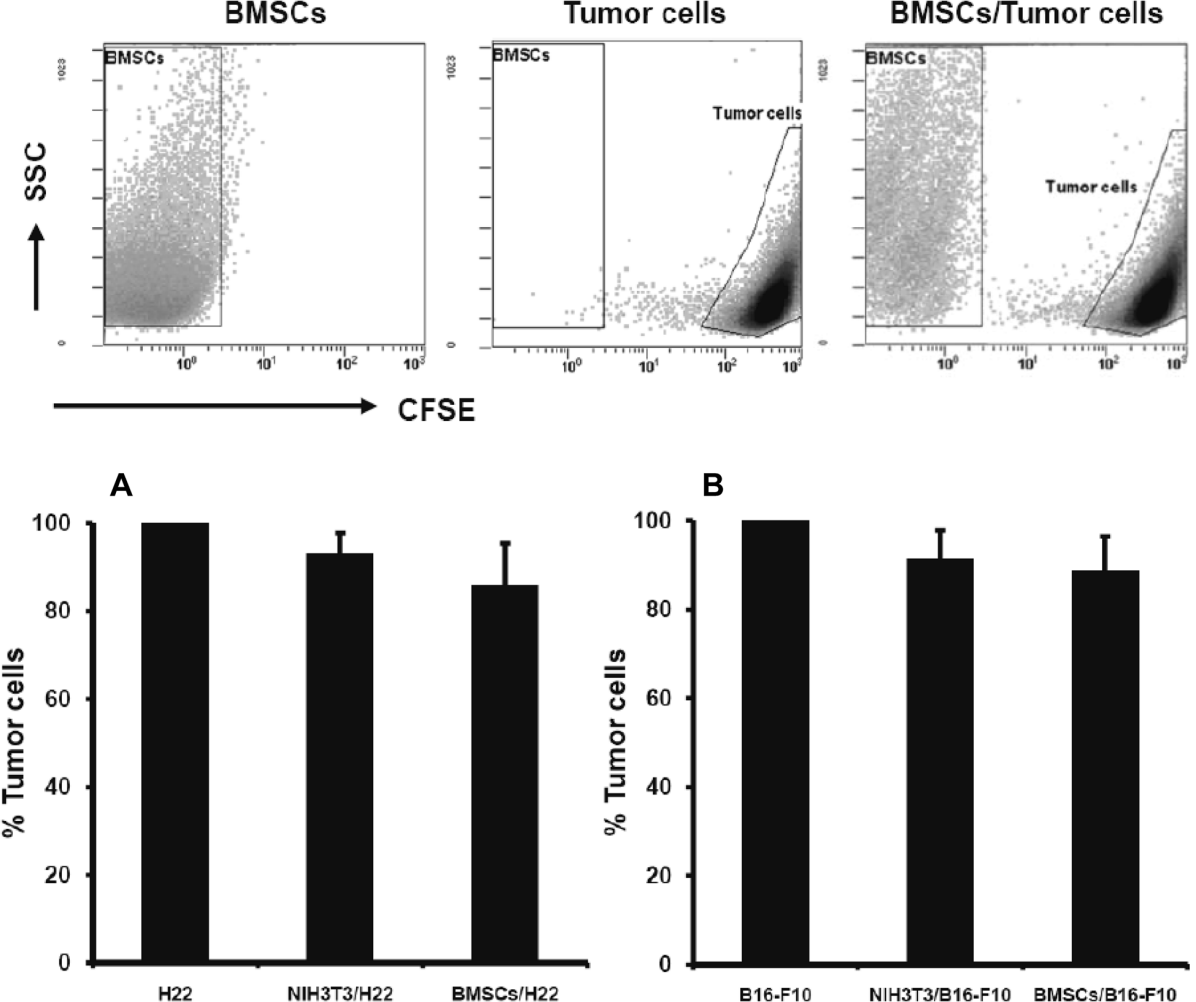
**Anti-tumor effect of bone marrow mesenchymal stem cells** ***in vitro*** **(** ***n*** **= 6).** Carboxy-fluorescein diacetate succinimidyl ester (CFSE)-labeled B16-F10 and H22 cells (2 × 10^5^ cells) were cultured either alone or in the presence of 2 × 10^5^ syngeneic bone marrow mesenchymal stem cells (BMSCs) or NIH-3T3 cells. Forty-eight hours after cultivation, the entire cell populations from individual culture or co-culture systems were harvested and the total number of CFSE^+^ tumor cells was calculated using flow cytometry. **(A)** H22/BMSC co-culture system. **(B)** B16-F10/BMSC co-culture system.

We wondered whether the injected BMSCs have a direct action in suppressing the proliferation of tumor cells via cell–cell contact manner. To check this hypothesis, carboxy-fluorescein diacetate succinimidyl ester-labeled H22 or B16-F10 cells were co-cultured with syngeneic BMSCs respectively at equal ratio for 48 hours. As a control, tumor cells were co-cultured with NIH-3T3 fibroblastic cells, to rule out a possible exhaustion of nutrients or a slowdown of growth due to a high density culture. Using flow cytometry analysis, we detected 11.4% and 8.6% B16-F10 cell reduction in the B16-F10/BMSC and B16-F10/NIH-3T3 co-culture systems respectively (*P* >0.05). Similar findings of 14.3% and 7.1% reduction in the number of H22 cells were observed in H22/BMSC and H22/NIH-3T3 co-culture systems respectively (*P* >0.05) (Figure 2). There was no statistical difference neither in the B16 nor in the H22 co-culture group.

### Bone marrow mesenchymal stem cells reduced Gr-1^+^CD11b^+^ MDSCs in tumor-bearing mice

**Figure 3:**
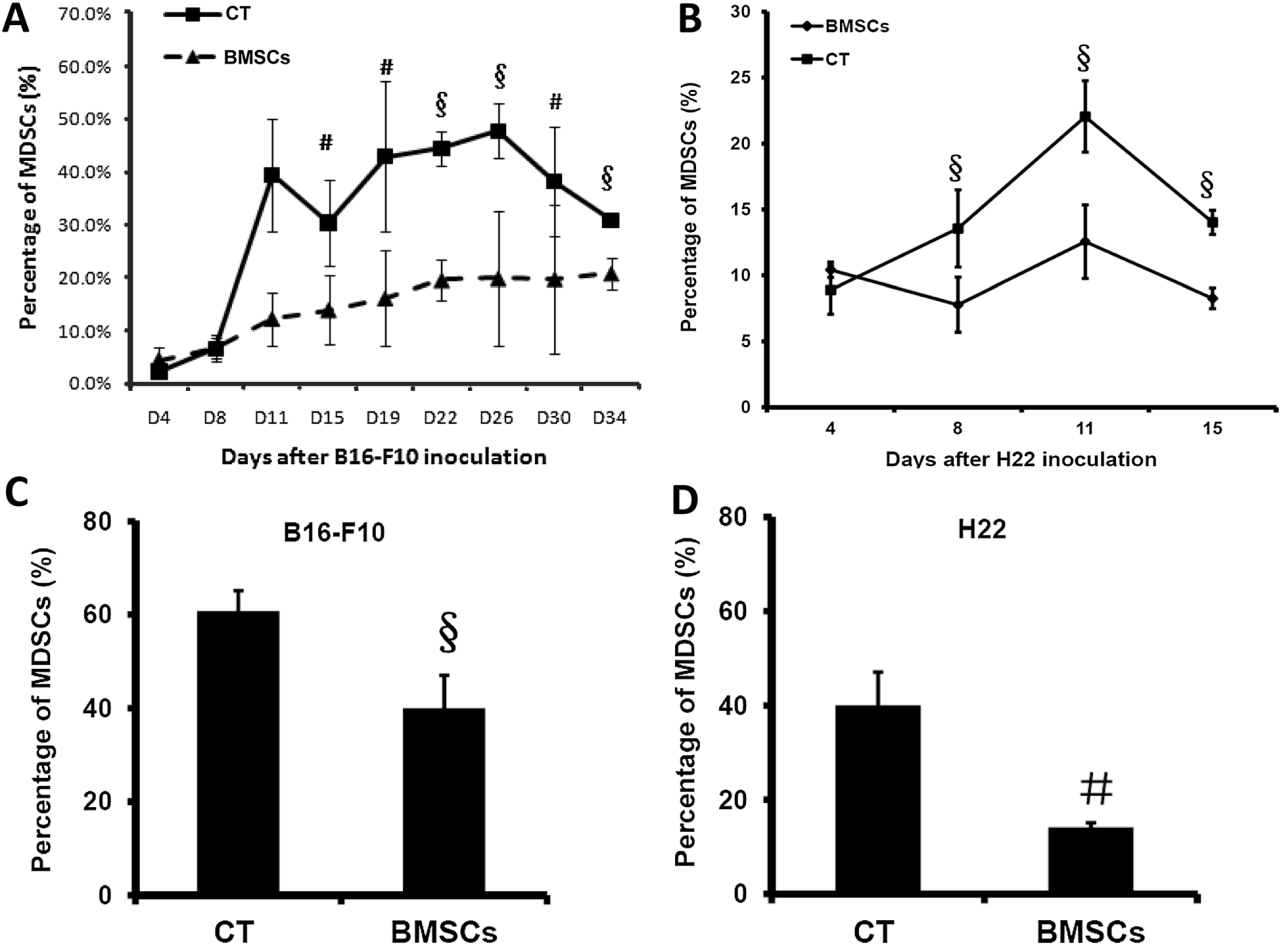
**Effect of bone marrow mesenchymal stem cells on Gr-1** ^**+**^ **CD11b** ^**+**^ **cells in tumor-bearing mice (** ***n*** **= 5).** Bone marrow mesenchymal stem cells (BMSCs) were injected through the tail vein in the B16-F10 pulmonary metastatic or H22 intraperitoneal hepatoma model and Gr-1^+^CD11b^+^ myeloid-derived suppressor cells (MDSCs) in peripheral blood and bone marrow were detected either during BMSC treatment or at the end of treatment. **(A)** Percentage of MDSCs in peripheral blood of C57 mice. **(B)** Percentage of MDSCs in peripheral blood of BALb/c mice. **(C)** Percentage of MDSCs in bone marrow of C57 mice. **(D)** Percentage of MDSCs in bone marrow of BALb/c mice. ^#^ *P* < 0.05; ^§^ *P* < 0.01 compared with the control (CT) group.

Aforementioned studies have shown that MDSCs could be induced and released from bone marrow in response to various stimuli, including cancer [21], and injected mouse BMSCs could relocate in bone marrow [10,23]. Therefore, we asked whether the induction and proliferation of an immunosuppressive cell subset of Gr-1^+^CD11b^+^ MDSCs could be affected following BMSC injection and homing to the bone marrow. To address this question, peripheral blood and bone marrow Gr-1^+^CD11b^+^ MDSCs were detected either every 4 days during BMSC treatment or at the end of treatment. As depicted in Figure 3, the percentage of Gr-1^+^CD11b^+^ MDSCs in peripheral blood from tumor-beating mice had a trend to increase with time during the tumor growth progressively, while the percentages of MDSCs were lower in the syngeneic BMSC-treated mice group compared with the control group. At the endpoint of tests, percentages of Gr-1^+^CD11b^+^ MDSCs in the bone marrow were analyzed by flow cytometry and the results showed a lower expression of MDSCs in the BMSC therapy groups, the differences between control and therapy groups being statistically significant (Figure 3C,D).

### Restoration of IFNγ^+^ T lymphocytes following BMSC transfusion in tumor-bearing mice

**Figure 4:**
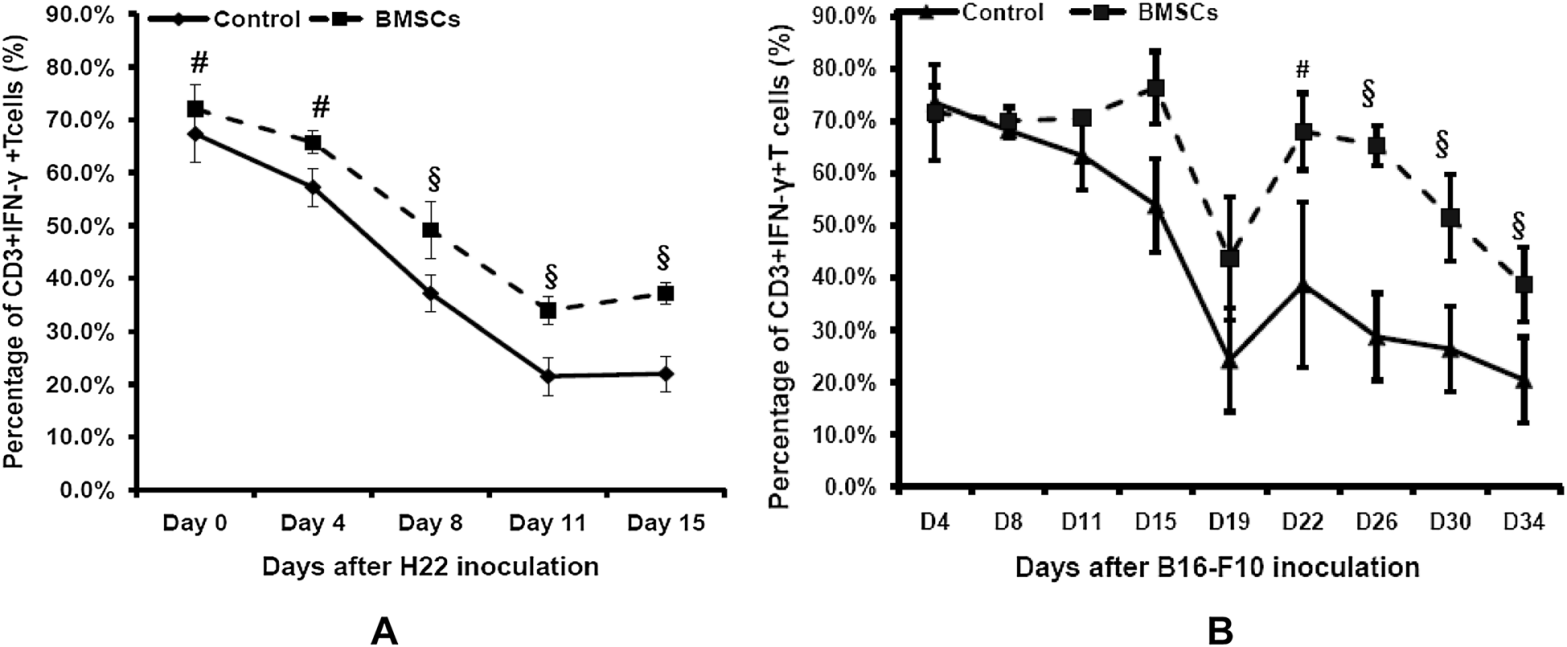
**Effect of bone marrow mesenchymal stem cells on IFNγ** ^**+**^ **T cells in tumor-bearing mice (** ***n*** **= 5).** After bone marrow mesenchymal stem cell (BMSC) treatment in the pulmonary metastatic or H22 intraperitoneal hepatoma model, IFNγ^+^ T cells in peripheral blood white cells were detected during and after BMSC treatment. **(A)** IFNγ^+^ T-cell percentage in peripheral blood white cells of C57 mice. **(B)** IFNγ^+^ T-cell percentage in peripheral blood white cells of BALb/c mice. ^#^ *P* <0.05; ^§^ *P* <0.01 compared with the control group. IFNγ, interferon gamma.

MDSCs suppress adaptive and innate immunity by inhibiting antigen-specific and nonspecific T-cell activation [21]. Since the induction and proliferation of the immunosuppressive cell subset of Gr-1^+^CD11b^+^ MDSCs were inhibited following BMSC injection and homing to the bone marrow, we also investigated activated subsets of T lymphocytes in tumor-bearing mice. The percentage of IFNγ^+^ T cells in peripheral blood is downregulated persistently during the tumor growth progressively among the B16-F10 pulmonary metastatic and H22 intraperitoneal hepatoma models, while in the BMSC-treated mice groups the percentages of IFNγ^+^ T cells were elevated about 15% (Figure 4). Therefore, it is reasonable to assume that BMSC treatment may reduce proliferation of MDSCs, which in turn maintains the IFNγ^+^ T cells in tumor-bearing mice.

### Bone marrow mesenchymal stem cells suppress MDSC induction and proliferation *in vitro*

**Figure 5:**
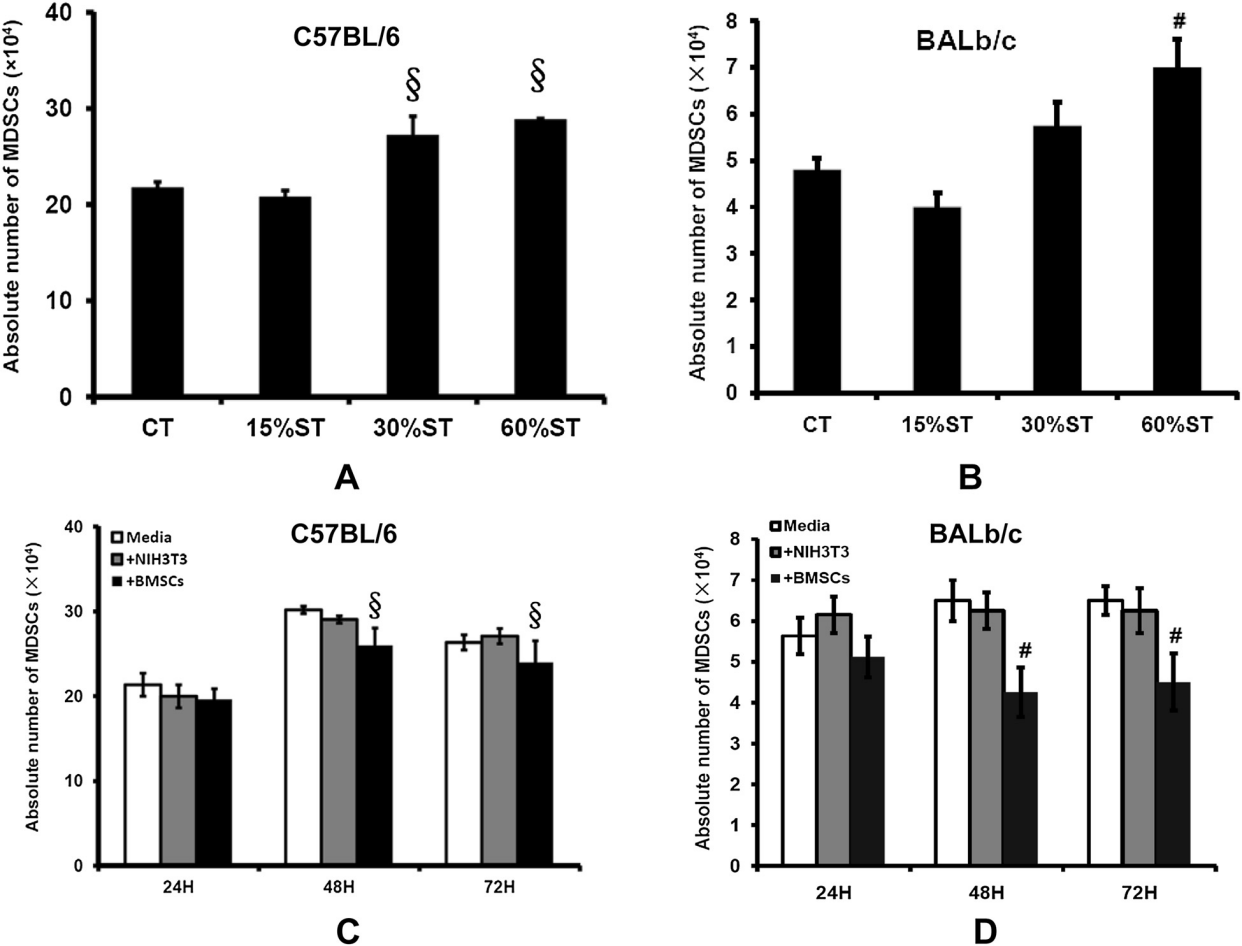
**Effect of bone marrow mesenchymal stem cells on myeloid-derived suppressor cells** ***in vitro*** **(** ***n*** **= 6).** Bone marrow cells (5 × 10^5^ cells) obtained from femurs and tibias of C57 and BALb/c mice were cultured in RPMI 1640 medium, alone, or in the presence of 10%, 30%, or 60% v/v tumor supernatant culture medium (ST), or co-cultured with 1 × 10^5^ syngeneic bone marrow mesenchymal stem cells (BMSCs). After 24, 48, and 72 hours, floating cells were gently collected and numbered. The absolute number of Gr-1^+^CD11b^+^ myeloid-derived suppressor cells (MDSCs) was calculated as described in Materials and methods. **(A)** Effect of BMSCs on MDSCs in the presence of B16-F10 culture medium. **(B)** Effect of BMSCs on MDSCs in the presence of H22 culture medium. **(C)** BMSC/bone marrow co-culture in the presence of B16-F10 culture medium. **(D)** BMSC/bone marrow co-culture in the presence of H22 culture medium. ^#^ *P* <0.05; ^§^ *P* <0.01 compared with the control (CT) group.

To confirm the *in vivo* inhibition of MDSCs by intravenous injected BMSCs, a series of bone marrow cell/BMSC co-culture experiments *in vitro* were performed to determine whether BMSCs could suppress MDSC induction and proliferation. First of all, tumor-conditioned media induced a proliferative response of MDSCs from either C57or BALb/c mice-derived bone marrow cells (Figure 5A,B). According to their effects, we had chosen 60% tumor condition medium for the following experiments. BMSCs caused a reduction of absolute MDSC number after 48 and 72 hours co-culture with bone marrow (Figure 5C,D). This finding was also proved in two types of syngeneic BMSCs and bone marrow cells separated from BALb/c and C57 mice, respectively.

### Co-culture with BMSCs decreases MDSC inhibition of CD3^+^IFNγ^+^ T cells *in vitro*

**Figure 6:**
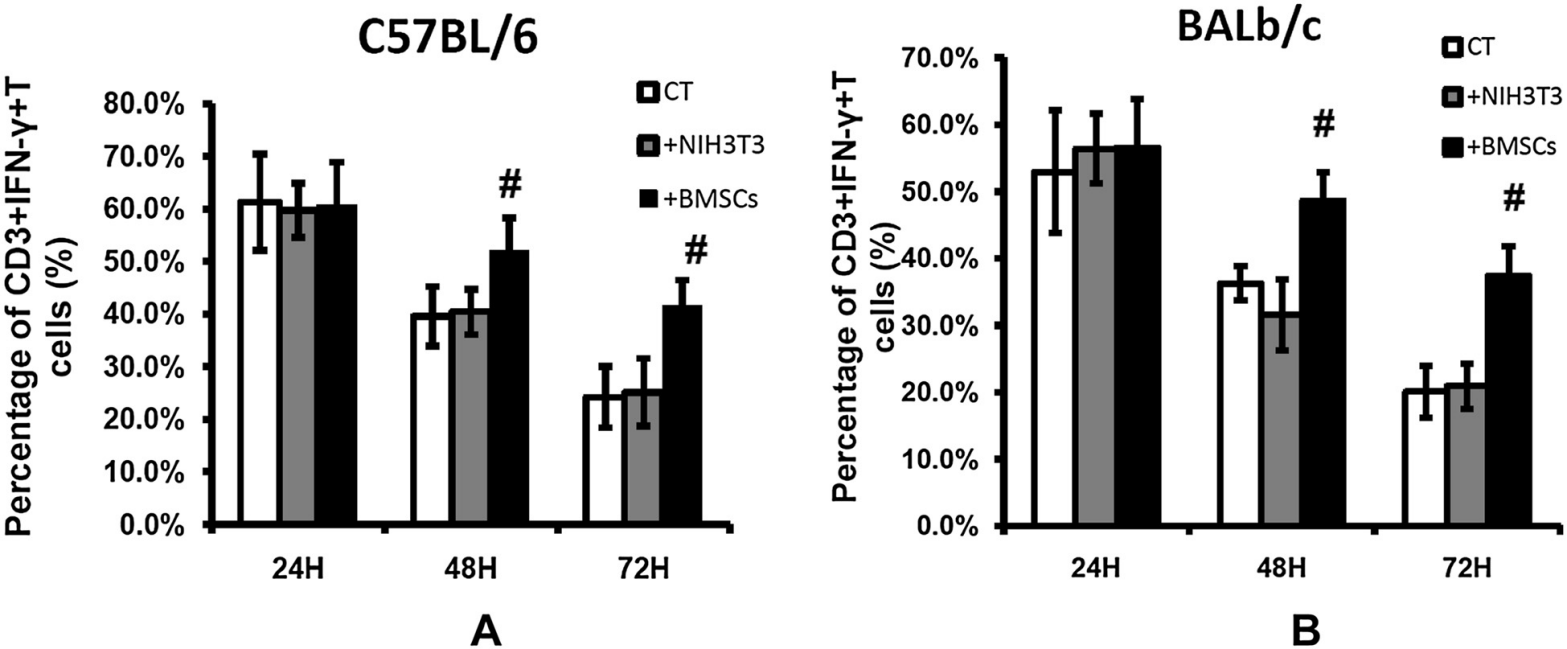
**Effect of bone marrow mesenchymal stem cells on CD3** ^**+**^ **IFNγ** ^**+**^ **T cells** ***in vitro*** **(** ***n*** **= 6).** Peripheral blood (PB) white cells (5 × 10^5^ cells) obtained from C57 and BALb/c mice were co-cultured with 1 × 10^5^ NIH-3T3 or syngeneic BMSCs in 60% v/v tumor supernatant culture medium with or without the presence of 1 × 10^5^ bone marrow suspension cells obtained from femurs and tibias of C57 and BALb/c mice. After 24, 48, and 72 hours, floating cells were gently collected and numerated. The percentage of CD3^+^IFNγ^+^ T cells in suspension cells was analyzed as described in Materials and methods. **(A)** Effect of BMSCs and MDSCs on PB white CD3^+^IFNγ^+^ T cells in the presence of B16-F10 culture medium. **(B)** Effect of BMSCs and MDSCs on PB white CD3^+^IFNγ^+^ T cells in the presence of H22 culture medium. ^#^ *P* <0.05. +BMSCs, PB white cells + MDSCs + BMSCs; CT, PB white cells + MDSCs; IFNγ, interferon gamma; +NIH3T3, PB blood white cells + MDSCs + NIH-3T3.

To observe the *in vitro* effect of BMSCs on CD3^+^IFNγ^+^ T cells, 5 × 10^5^ peripheral blood white cells obtained from C57 and BALb/c mice were co-cultured with 1 × 10^5^ NIH-3T3 or syngeneic BMSCs in 60% v/v tumor supernatant culture medium with or without the presence of 1 × 10^5^ bone marrow suspension cells (MDSCs) obtained from femurs and tibias of C57 and BALb/c mice. After 24, 48, and 72 hours, floating cells were gently collected and numerated. The percentage of CD3^+^IFNγ^+^ T cells in the suspension cells was analyzed by flow cytometry We did not observe any difference in CD3^+^IFNγ^+^ T-cell percentage among the three groups (peripheral blood white cell group, peripheral blood white cells co-cultured with NIH-3T3, or BMSCs) without the presence of bone marrow suspension cells (data not shown). When MDSCs presented, the percentage of CD3^+^IFNγ^+^ T cells was higher in the BMSC groups after 48 hours and 72 hours co-culture for both B16-F10 and H22 cells (Figure 6). These differences were statistically significant (*P* <0.05).

## Discussion

Although various studies have shown that MSCs may modulate the growth of tumor cells, the observed discrepancies probably reflect the differences in the experimental settings. The origin of the MSCs, the strain of the mouse, the type of tumor cells, or the method of treatment might interfere with the behavior of the tumor [24]. BMSCs are able either to favor angiogenesis and tumor initiation or to inhibit progression of established tumors [17]. The present study aimed at evaluating the effect of BMSCs on the development and progression of metastatic tumors in syngeneic immunocompetent animals. On this account, we used two metastatic tumor models –ascitogenous hepatoma in the BALb/c mouse and B16-F10 pulmonary metastatic melanoma in the C57 mouse – which may help to understand whether the systemic injection of MSCs in a therapeutic setting might influence the period of tumor growth and metastasis. Our results showed that, no matter whether in hepatoma-burdened BALb/c mouse or in melanoma-burdened C57 mouse, BMSC therapy improved mice lifetime and retarded the development of metastatic tumor, which is coincident with other research [25]. However, we also noticed that there were contradictory results about the activity of MSCs promoting tumor cell growth. MSCs might have two different potentials to support or suppress tumor growth [26]. Their function in the tumor microenvironment and the mechanism of their functioning need more research and illustration.

Previous studies suggest that systemically infused BMSCs have the ability of homing to bone marrow and sites of active tumorigenesis. Although it was reported that only 0.56 to 0.72% of BMSCs home to tumor mass [27], BMSCs still have the possibility to exert an anti-tumor effect by direct cell–cell contact. Roodra and colleagues have shown that MSCs provide direct cell–cell contact interactions and effect tumor growth by soluble factor secretion [28]. To rule out a possible direct effect of BMSCs on tumor cell proliferation, we cultured tumor cell lines in the presence of BMSCs or NIH-3T3 fibroblastic cells *in vitro*. The presence of BMSCs had no effect on the growth of B16-F10 and H22 tumor cells *in vitro*. Heil and colleagues have shown that BMSCs promote neoangiogenesis through paracrine secretion [29]. We might suppose that BMSCs probably act through paracrine secretion or a systemic regulation rather than locally, even though complementary *in vitro* and *in vivo* experience seems more persuasive than *in vitro* experience only. The microenvironment of a progressing tumor is composed of proliferating malignant cells, blood vessels, tumor stroma, and infiltrating inflammatory cells. This is a unique environment dominated and created by tumor cells that establish specific interactions with neighbor cells in order to promote tumor progression and metastasis. Moreover, the immune system of our body is a complex network. With these two complicated systems, the tumor-dominated microenvironment and the immune system, it is difficult to explain the numerous conflicting reports on the effects of MSCs on tumors. Therefore, it is important to further explore the interaction of MSCs with tumor cells in the tumor microenvironment and in the whole immune system.

MSCs are firstly thought to have immunosuppressive effects and to promote both hematopoietic and solid organ transplantation tolerance [30], which may be an important reason why they are thought to increase incidence of tumor formation or promote tumor growth *in vivo*. Their immune-modulating effects have been explored deeper over time, however, and BMSCs have been found to drive immune responses in different ways according to the microenvironment provided to them [31]. As a member of the immune cells, MDSCs are a heterogeneous population cells of myeloid origin including myeloid progenitors and immature myeloid cells. In healthy individuals, immature myeloid cells are mobilized from bone marrow and differentiate into mature dendritic cells, macrophages, and granulocytes. In contrast, in pathological conditions such as cancer, immature myeloid cells are blocked to differentiate into mature myeloid cells, and MDSC activation and expansion occur. Like MSCs, MDSCs can be generated from bone marrow into the bloodstream and home in tumor stroma, where they promote tumor growth [32]. Thus, we suppose that BMSCs might modulate tumor growth through immune systemic regulation; it is possible that they improve or inhibit some of the immune cells, including MDSCs. To prove this hypothesis, we detected the percentage of Gr-1^+^CD11b^+^ MDSCs in peripheral blood and bone marrow during or at the end of BMSC treatment. As the aforementioned results have shown, compared with control groups MDSCs presented a larger quantity in peripheral blood and bone marrow in BMSC-treated tumor-bearing mice. This property was further defined by using an *in vitro* MDSC-inducing system, which demonstrated significant suppressive effect of BMSCs on the MDSC proliferation. We may conclude that persistent administration of BMSCs is able to suppress metastasis tumor progression, and MDSCs might take a part of role in this effect, at least in the experimental conditions tested here.

In our experiments, there was also an upregulation of IFNγ^+^ T cells with the tumor growing time in the BMSC-treated tumor-bearing model. Our *in vitro* experiment confirmed this result, which showed that after peripheral blood white cells were co-cultured with NIH-3T3 or BMSCs in tumor supernatant without MDSCs there was no difference in the percentage of CD3^+^IFNγ^+^ T cells. When MDSCs were present, however, the percentage of CD3^+^IFNγ^+^ T cells was higher than control in BMSC groups after 48 hours and 72 hours co-culture for both B16-F10 and H22 cells. We can conclude that an MDSC presence affects the number of CD3^+^IFNγ^+^ T cells in the tumor microenvironment *in vitro*. Several investigators have demonstrated that MDSCs mediated an inhibiting function of antigen-specific and nonspecific T-cell activation. Lesokhin and colleagues showed that MDSCs are important contributors to the development of an immunosuppressive tumor microenvironment that blocks the action of cytotoxic anti-tumor T-effector cells. MDSCs regulate entry of activated CD8^+^ T cells into the tumor site, thereby limiting the efficacy of immunotherapy [33]. An anti-interleukin-6R monoclonal antibody could downmodulate MDSCs in tumor-bearing mice, which enhance anti-tumor T-cell responses and lead to an anti-tumor effect [34]. MDSCs may inhibit T-cell activity by nitric oxide production [35], 5-fluorouracil [36] and docetaxel application through inhibition of the STAT3 pathway [37]. Adverse opinion is that BMSCs can also directly modulate T cells and other immune cells [38]. Necessary follow-up work should be designed to test whether this effect is triggered by a direct modulation of BMSCs or by MDSCs.

In summary, BMSCs have a potential anti-tumor effect in our metastatic tumor model therapy. In our study, BMSCs downmodulated MDSCs in peripheral blood and in bone marrow. This might be one of the mechanisms by which BMSCs inhibit metastatic tumor. However, the issue of whether BMSCs can be used as a therapeutic strategy requires further investigation into safety and clinical evaluation of BMSCs, illustration of their mechanisms in immune modulation, and interactions between BMSCs and tumor cells in a tumor-dominant microenvironment.

## Conclusion

BMSC treatment demonstrated anti-tumor function presenting as mice lifetime extension and inhibition of metastatic tumor growth. BMSCs modulating the formation and maturity of MDSCs might be one of the mechanisms to explain this tumor-suppressive effect. Decrease of CD3^+^IFNγ^+^ T cells in the tumor microenvironment can be partially converted by BMSCs *in vivo* and this effect might be related to MDSCs. BMSC treatment may present a potential therapy in cancer treatment after deep research in the clinical practices performed.

## Abbreviations

BMSC: bone marrow mesenchymal stem cell
Gr-1(Ly-6G): lymphocyte antigen 6 complex, locus G
IFNγ: interferon gamma
MDSC: myeloid-derived suppressor cell
MSC: mesenchymal stem cell
PBS: phosphate-buffered saline
